# Temperature Dependent Micro-Structure of KAlF_4_ from Solid to Molten States

**DOI:** 10.3390/ma11101846

**Published:** 2018-09-27

**Authors:** Nan Ma, Jinglin You, Liming Lu, Jian Wang, Songming Wan

**Affiliations:** 1 State Key Laboratory of Advanced Special Steel, Shanghai Key Laboratory of Advanced Ferrometallurgy, School of Materials Science and Engineering, Shanghai University, Shanghai 200444, China; doria_mn@163.com (N.M.); wj581692@126.com (J.W.); 2 CSIRO Mineral Resources, Technology Court, Pullenvale, QLD 4069, Australia; liming.lu@csiro.au; 3 Anhui Key Laboratory for Photonic Devices and Materials, Anhui Institute of Optics and Fine Mechanics, Chinese Academy of Sciences, Hefei 230031, China; smwan@aiofm.ac.cn

**Keywords:** KAlF_4_, in situ, layered structure, [AlF_6_]^3−^ octahedra, [AlF_4_]^−^ tetrahedra

## Abstract

In situ high temperature X-ray diffraction and Raman spectroscopy were used to investigate the temperature dependent micro-structure of KAlF_4_. Density functional theory was applied to simulate the structure of crystalline KAlF_4_ while a quantum chemistry ab initio simulation was performed to explore the structure of molten KAlF_4_. Two crystal polymorphs demonstrated to be present in solid KAlF_4_. At the temperature below 673 K, it belongs to the tetragonal crystal system within the P4/mbm space group, while the high temperature phase is attributed to the monoclinic crystal system within the P2_1_/m space group. Both polymorph KAlF_4_ phases are characterized by a layered structure consisting of K^+^ and [AlF_6_]^3−^ octahedra, each of the [AlF_6_]^3−^ octahedra equivalently shares four corners with other four [AlF_6_]^3−^ octahedra along the layer. The layered structure became unstable at higher temperatures and crashed when the temperature exceeded the melting point. It demonstrated that the molten KAlF_4_ consisted of predominant [AlF_4_]^−^ and a small amount of [AlF_6_]^3−^. The Raman spectrum of molten KAlF_4_ simulated by using a quantum chemistry ab initio method agreed well with the experimental Raman spectrum.

## 1. Introduction

The physicochemical properties of molten fluoroaluminates are of considerable importance for the aluminum industry. Molten KAlF_4_ had been employed not only as electrolyte in producing aluminum [1] but also as a basic part of the flux in the aluminum brazing technology [2]. Hence an understanding of the molten structure of KAlF_4_ is essential for obtaining necessary knowledge of the properties of the electrolytes for aluminum smelters and the fluxing agent for the aluminum brazing industry.

Over the years, limited work has been carried out focusing on the structure feature of molten KAlF_4_. The room temperature phase [3,4,5], the structure from the low temperature (4 K) to room temperature [6,7] and the molten states [8] of KAlF_4_ were extensively investigated. According to Chen’s work [9,10], congruent melting occurred at 575 °C when KAlF_4_ was heated. The KAlF_4_ crystal consists of six-coordinated aluminum atoms, which are linked to four bridging fluorine ions, two non-bridging fluorine ions and potassium ions [7,11,12]. The main vibrational wavenumber of molten NaAlF_4_ at about 630 cm^−1^ was detected by Robert [13] by using high temperature Raman spectroscopy. With the consideration of the results by high temperature NMR spectra, the band at Raman wavenumber of 630 cm^−1^ was further assigned to the symmetric stretching vibrations of the [AlF_4_]^−^ tetrahedron. Akdeniz concluded that the Raman peaks observed at 621 cm^−1^ for NaAlF_4_ are related to the Al-F bond of isolated [AlF_4_]^−^ clusters with a bond length of 1.69 Å [14], as was also assured by molecular simulation [15]. Due to the high corrosivity of KAlF_4_ melt, the structure evolution from room temperature to the molten states of KAlF_4_ has not been cleared.

In the present paper, temperature dependent Raman spectroscopic studies of the micro-structure of KAlF_4_ up to the molten state were conducted in conjunction with high temperature X-ray diffraction to explore the structure evolution of KAlF_4_ from room temperature to above its melting point. Raman spectra simulations based on density functional theory (DFT) and quantum chemistry ab initio methods were also applied to make the assignments of the characteristic vibration modes of Raman spectra observed at different temperatures.

## 2. Materials and Methods

### 2.1. Material Synthesis

All chemicals including AR grade K_2_CO_3_, Al(OH)_3_, and other reagents used in the present study, were obtained from Sinopharm Chemical Reagent Co., Ltd. K_2_CO_3_ and Al(OH)_3_ were first dried at 473 K for 2 h to remove the surface moisture. Stoichiometric amounts of K_2_CO_3_ and Al(OH)_3_ were then mixed with an excess amount of 40% hydrofluoric acid in a teflon cup to form a paste. The paste was dried at 373 K in a platinum crucible. After being ground in an agate mortar, the dried mixture was annealed in a platinum crucible at 723 K for 48 h [9]. The resultant product was ground for subsequent experiments.

### 2.2. Material Characterization

In situ high temperature X-ray powder diffraction studies were conducted using the Bruker D8 Advance X-ray diffractometer (40 KV, 40 mA) with Cu/Kα1 radiation (λ = 0.15406 nm). The patterns were recorded in an inert atmosphere in the 2θ range of 10 to 100° with a step size of 0.02° and a counting time of 0.33 s per step.

In situ high temperature Raman spectroscopic studies were carried out under a dry inert atmosphere using the LabRAM HR800 Raman spectrometer (Horiba Jobin Y’von, Paris, France) equipped with an ultraviolet pulse laser beam of 355 nm which was focused on the sample through a microprobe with a 4× objective lens. The average laser beam power on the sample was about 60 mW. It was equipped with a microscopic heating furnace (TS1500) (Linkam, Tadworth, UK) with a small temperature deviation of about ±1 K for investigating the microstructure of the sample at different temperatures. The platinum crucible which was applied to measure the high temperature experiment is protected by a patent [16]. A charge coupled device (CCD) detection system by an accumulated mode of 20 × 20 (20 times with 20 s each time) was used to collect Raman scattering light. The sample was held at the targeted temperature for 5 min before recording Raman spectra in order to ensure the sample reaches the targeted temperature and also avoids volatilization of the sample.

### 2.3. Computational Details

By using the functional set GGA [17] (WC [18]) version with optimized norm-conserving pseudopotentials [19], CASTEP (Cambridge Serial Total Energy Package [20,21]) based on DFT with the plane-wave pseudopotential method [22], was applied to calculate vibrational modes of KAlF_4_. The plane-wave cut-off energy was fixed at 780.0 eV. The other parameters which were set in the computation, included a grid of 3 × 2 × 2 k-points and a self-consistent field (SCF) convergence threshold of 2 × 10^−6^ eV/atom.

In order to examine the clusters existing in the molten KAlF_4_, a simulation based on a quantum chemistry ab initio method was also carried out. A series of aluminum fluoride model clusters with typical characteristic structures were proposed and optimized for geometric configuration before the simulation. The restricted Hartree-Fock (RHF) [23] calculation method with a 6-31G (d) [24] basis set was applied for geometry optimization and the calculation of the vibration frequencies of various molecules.

## 3. Results and Discussion

### 3.1. Room Temperature Characteristics of KAlF_4_

**Figure 1:**
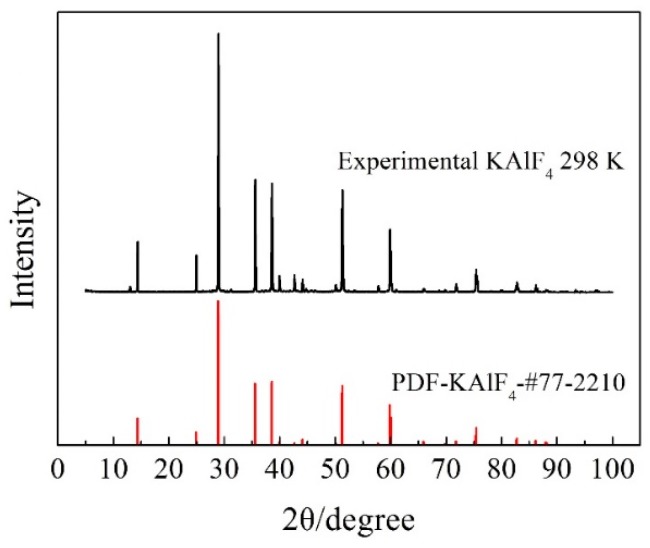
Room temperature XRD pattern of the synthesized KAlF_4_ compound. The PDF card No.77-2210 was obtained from the JCPD (Joint Committee for Powder Diffraction Standards) database for synthetic KAlF_4_.

The XRD pattern of the synthesized KAlF_4_ compound is shown in Figure 1. Both the peak position and relative intensity of main diffraction peaks, were in good agreement with the information provided in the powder diffraction file (PDF) data base for KAlF_4_, confirming the dominance of the KAlF_4_ compound in the sample.

**Figure 2:**
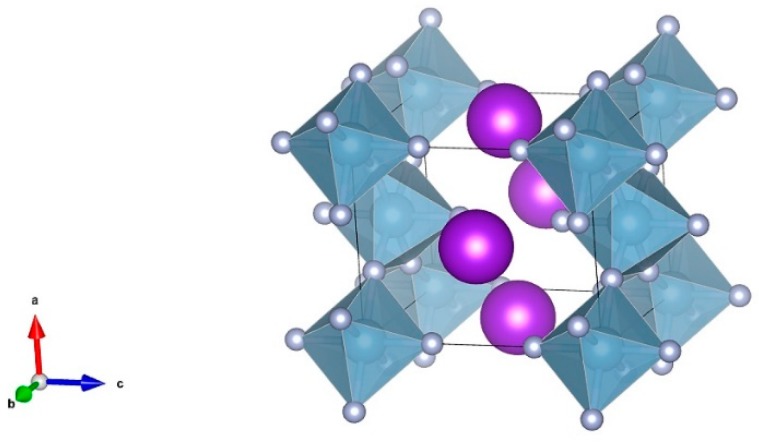
The unit cell structure of the room temperature KAlF_4_ crystal. The purple balls represent the K atoms while the dark cyan octahedral structures represent the [AlF_6_]^3−^ octahedra.

At the ambient temperature and pressure, the KAlF_4_ phase is characterized by a layered structure consisting of K^+^ and [AlF_6_]^3−^ octahedra, each [AlF_6_]^3−^ octahedron equivalently shares four corners with other four [AlF_6_]^3−^ octahedra within the layer. The [AlF_6_]^3−^ octahedron consists of a six-coordinated aluminum atom which is linked to six fluorine ions including four bridging fluorine ions and two non-bridging fluorine ions. Figure 2 illustrates a unit cell structure of the room temperature KAlF_4_ crystal, drawn by the VESTA software [25]. The room temperature KAlF_4_ unit cell is tetragonal with dimensions a = 5.122 Å, b = 5.122 Å, c = 6.288 Å, α = β = γ = 90°, V = 164.999 Å^3^, belonging to the P4/mbm space group [3].

**Figure 3:**
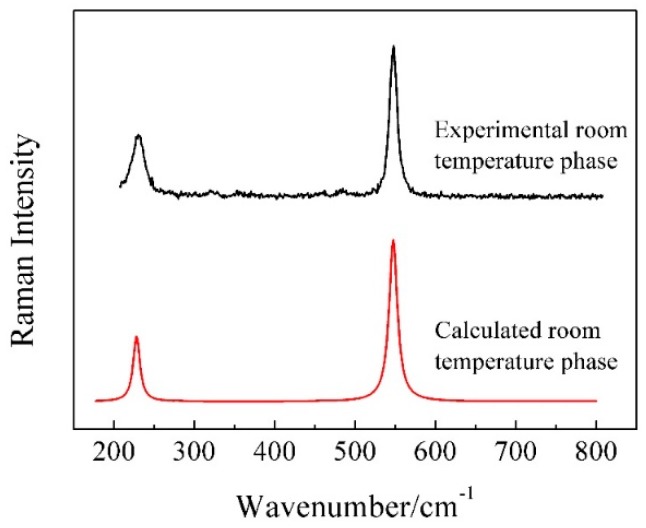
The room temperature Raman spectrum of the KAlF_4_ crystal is compared with the calculated (using CASTEP) followed by a Raman shift correction with a factor of k_1_ = 0.000426*x* + 0.80 and the Lorentzian smearing is 10 cm^−1^.

The room temperature Raman spectrum was calculated by CASTEP for the KAlF_4_ crystal and is compared in Figure 3 with the experimental spectrum obtained for the synthesized KAlF_4_ compound. The small shift in wavenumbers between the experimental and calculated spectra is due to the precision and limitation of the theoretical simulation methods. In order to compare with the experimental spectrum, the calculated spectrum was correct by a factor k_1_ = 0.000426*x* + 0.80, where the factor k_1_ is a function of wavenumber *x*.

In Figure 3, there are two well defined peaks in the calculated spectrum, which locates at 227.6 and 547.4 cm^−1^. The calculation results show that the band at 227.6 cm^−1^ relates to the shearing vibrations of Al^VI^-F_nb_ (F_nb_ represents non-bridging fluorine) while the band at 547.4 cm^−1^ corresponds to the symmetric stretching vibrations of Al^VI^-F_nb_. According to the group theory analysis, the vibrational modes are distributed among the following irreducible representations: 4*A*_2*u*_ + 16*E_u_* + 2*B*_2*u*_ + 2*A*_1*g*_ + 6*E_g_* + 1*B*_2*g*_ + 2*A*_1*u*_ + 2*A*_2*g*_ + 1*B*_1*g*_, where only *A*_1*g*_, *E_g_*, *B*_2*g*_ and *B*_1*g*_ are Raman-active modes based on the selection rules. Similarly, two peaks were observed at 228 and 547 cm^−1^ in the wavenumber range of 200–600 cm^−1^ of the experimental spectrum at room temperature. Comparing with the calculated results of the room temperature KAlF_4_ crystal, the band at 228 cm^−1^ is believed to be due to the shearing vibrations of Al^VI^-F_nb_, while the band at 547 cm^−1^ results from the symmetric stretching vibrations of Al^VI^-F_nb_. Overall, the simulated results were in good agreement with the experimental spectra for the main vibration peaks. Therefore, it is feasible to apply DFT to calculate Raman spectra of fluoroaluminate systems [26,27]. The major vibration modes of the room temperature phase of KAlF_4_ are listed in Table 1. And all of the calculated Raman modes of the room temperature phase of KAlF_4_ are shown in Table S1 (in Supplementary Materials).

### 3.2. The Phase Transformation of KAlF_4_

**Figure 4:**
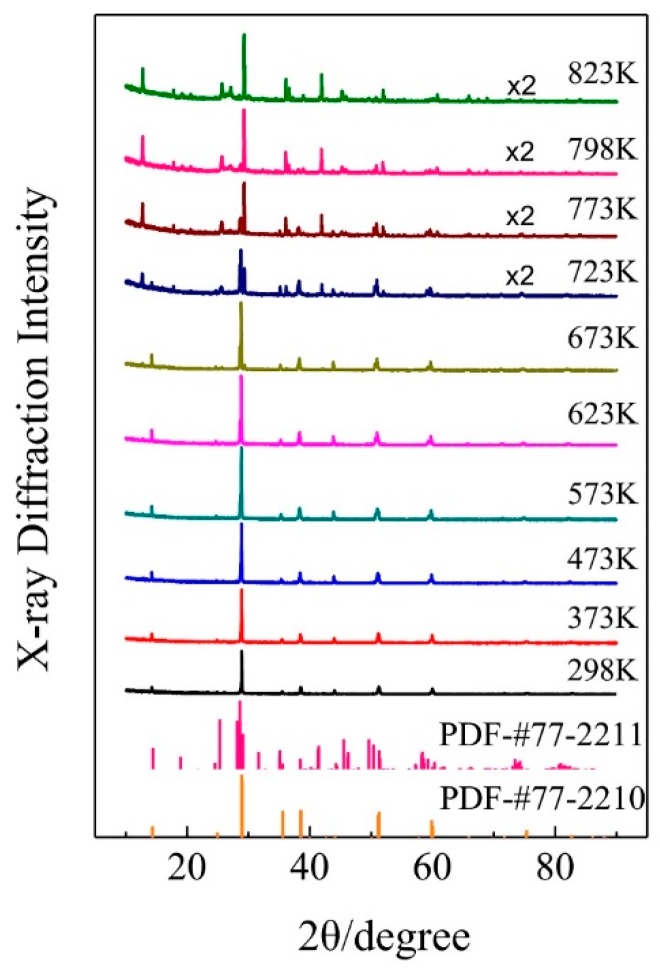
Temperature dependent X-ray diffraction spectra collected from room temperature up to 823 K for the synthesized KAlF_4_ compound. No.77-2210 and 77-2211 were obtained from the JCPD (Joint Committee for Powder Diffraction Standards) database for the tetragonal and monoclinic KAlF_4_ unit cell structures, respectively.

The temperature dependent X-ray diffraction spectra of the synthesized KAlF_4_ compound were recorded while the sample was heated from room temperature to 823 K. The sample was heated at about 10 K/min under an argon atmosphere and held at each temperature point for five minutes prior to measurement to ensure that the sample reached the designated temperature. As shown in Figure 4, the sample maintained in its room temperature tetragonal structure until 673 K and then transformed to the high temperature phase as the sample temperature was further raised from 673 to 723 K. The high temperature phase of KAlF_4_ is characterized by a monoclinic structure, belonging to the P2_1_/m space group, with its unit cell dimensions a = 6.542 Å, b = 7.195 Å, c = 7.177 Å, α = 90°, β = 108.478°, γ = 90°, V = 320.415 Å^3^, which is similar to the structure in the literature [7].

**Figure 5:**
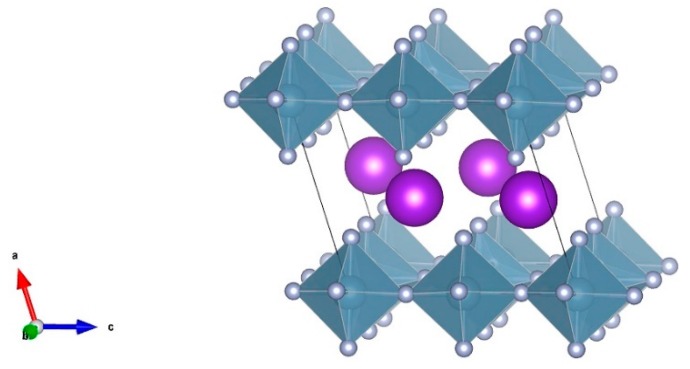
The unit cell structure of high temperature phase of KAlF_4_. The purple balls represent K ions, while the dark cyan octahedral structures represent [AlF_6_]^3−^ octahedra.

Figure 5 shows a monoclinic unit cell structure of the high temperature phase of KAlF_4_ which consists of potassium ions and a six-coordinated Al atom forming an octahedron. The high temperature phase was also characterized by a layered structure consisting of K^+^ ions and [AlF_6_]^3−^ octahedra, where each [AlF_6_]^3−^ octahedron shares four corners with other [AlF_6_]^3−^ octahedra within the layer. The potassium ions between the [AlF_6_]^3−^ octahedron layers were used to keep the charge balanced. Comparing with the room temperature phase, the structure of the high temperature phase is more distorted due to the temperature impact, resulting in a more complicated XRD spectrum, as evidenced in Figure 4.

**Figure 6:**
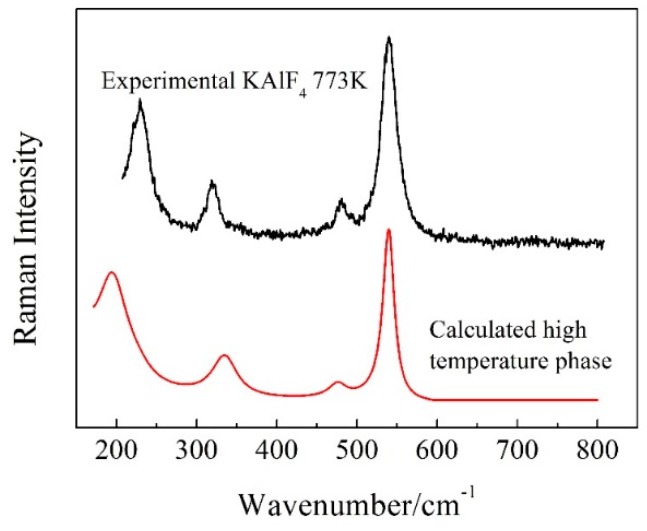
The Raman spectrum of the high temperature phase of KAlF_4_ calculated by CASTEP (using a Raman shift correction with a factor of k_2_ = −0.000835*x* + 1.41) and the experimental Raman spectrum of the same phase at 773K. The Lorentzian smearing is 40 cm^−1^.

The Raman spectrum of the high temperature phase of KAlF_4_ was calculated (based on its cell structure in Figure 5) by CASTEP and is shown in Figure 6 together with the experimental spectrum obtained at 773 K. The calculated wavenumber values of the high temperature phase of KAlF_4_ were corrected with a factor k_2_ = −0.000835*x* + 1.41 to account for the shifts for comparing with the experimental spectrum. Overall, there were very little distinction between the simulated and experimental spectra for the main vibrational peaks. As shown in Figure 6, four peaks, located at 193.9, 335.9, 474.5 and 539.5 cm^−1^, respectively, were identified from the calculated spectrum. Based on simulation, the bands at 193.9, 335.9 and 474.5 cm^−1^ are assigned to the shearing vibrations of Al^VI^-F_nb_, while the Raman band at 539.5 cm^−1^ is assigned to the symmetric stretching vibrations of Al^VI^-F_nb_. According to the group theory analysis, the vibrational modes are distributed among the following irreducible representations: 13*B_g_* + 19*A_u_* + 23*B_u_* + 17*A_g_*, where only *A_g_* and *B_g_* are Raman-active modes based on the selection rules. The major vibration modes calculated for the high temperature phase of KAlF_4_ are listed in Table 2. And all of the calculated Raman modes of the high temperature phase of KAlF_4_ are shown in Table S2 (in Supplementary Materials). Similar peaks were observed at 229, 319, 481 and 539 cm^−1^ in the 773 K experimental spectrum. Comparing with the calculated spectrum of the high temperature phase of KAlF_4_, the bands at 229, 319 and 481 cm^−1^ are believed to represent the shearing vibration of Al^VI^-F_nb_ and the band at 539 cm^−1^ is relating to the symmetric stretching vibrations of Al^VI^-F_nb_. Two new bands which are located at 319 and 481 cm^−1^, were observed at 773 K, are related to the transformation of the unit cell structure from tetragonal to monoclinic.

**Figure 7:**
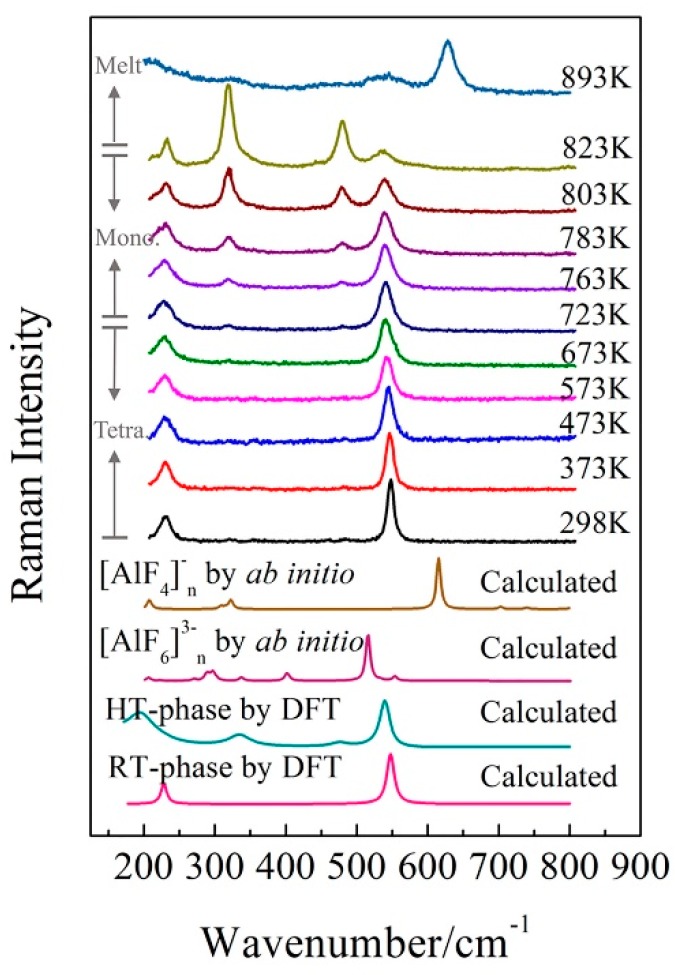
Temperature dependent Raman spectra of KAlF_4_ and the Raman spectra calculated by DFT and an ab initio method. The value of n is equal to 7 in the calculated [AlF_4_]^−^_n_ by ab initio. The calculated [AlF_6_]^3−^_n_ by ab initio is referred by our previous work [28].

Figure 7 presents the Raman spectra of the KAlF_4_ crystal recorded when the sample was heated (at a heating rate of 10 K/min) from room temperature to 823 K and at a heating rate of 5 K/min afterwards. The high temperature spectra were recorded 5 min after the sample reached the target temperature. Figure 7 includes the calculated Raman spectrum of [AlF_6_]^3−^_n_ clusters (by ab initio method) which refers to our previous work [28]. As the sample temperature increased, the Raman band at 547 cm^−1^ shows a clear evidence of band broadening and redshifts in wavenumber.

**Figure 8:**
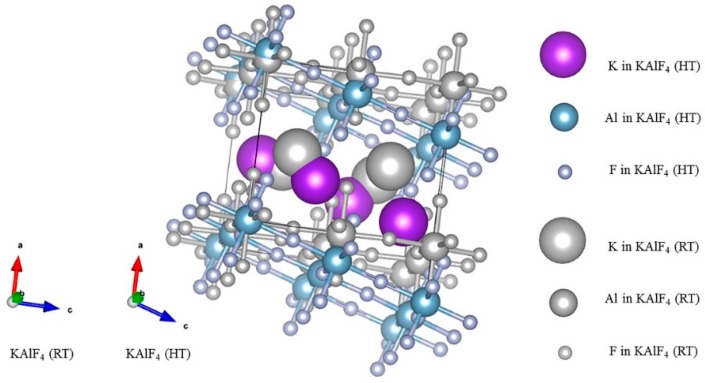
Intermediate KAlF_4_ structure as it evolves from the room temperature to high temperature structure.

Two new Raman bands at 319 and 481 cm^−1^ started to appear at 673 K and were gradually enhanced as the sample temperature increased from 673 to 823 K. As discussed earlier, the bands at 319 and 481 cm^−1^ were attributed to the shearing vibrations of Al^VI^-F_nb_ of the [AlF_6_]^3−^ octahedra with the high temperature phase. As clearly shown in Figure 4, the sample transformed from the tetragonal to monoclinic structure as the sample temperature was further raised from 673 to 723 K. Table 3 summarizes the structure parameters of the room and high temperature phases. Figure 8 illustrates an intermediate KAlF_4_ structure as it evolves from the room to high temperature structure. The room temperature and high temperature phase are drawn in a simple diagram. In Figure 8, the grey unit cell represents the room temperature phase while the chromatic unit cell constitutes the high temperature phase. As KAlF_4_ turns from the tetragonal to monoclinic structure approximately at 723 K, the space group changes from P4/mbm to P2_1_/m. The cell length becomes much longer, and one of the cell angles increases from 90.000 to 108.478°. As a consequence, the distribution of the bond length and bond angle of KAlF_4_ becomes more complicated.

As the sample temperature increased further, the sample was fully melted at 893 K and is characterized by the molten structure as evidenced in Figure 7.

### 3.3. Characteristics of Molten KAlF_4_

**Figure 9:**
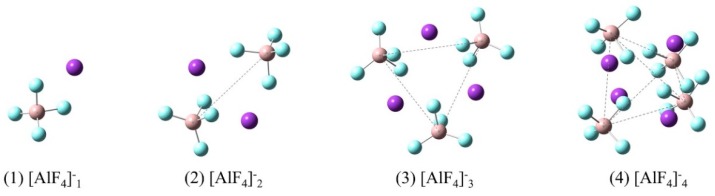
Model clusters used for quantum chemistry ab initio methods: [AlF_4_]^−^ denotes an ionic molecule consisting of a four-coordinated Al atom and four F atoms while n (=1,2,3,4) stands for the number of [AlF_4_]^−^ molecules in the clusters. The purple, beige and cyan balls represent K, Al and F atoms, respectively. The spatial configurations of the central atoms of [AlF_4_]^−^ in the cluster are point, linear, triangular and tetrahedronal respectively from n = 1 to 4.

According to the literatures [29,30], [AlF_4_]^−^ is the main species in molten KAlF_4_. Figure 9 illustrates a series of possible [AlF_4_]^−^_n_ model clusters presented in molten KAlF_4_. After being optimized for geometry configuration, these model clusters were used for a quantum chemistry ab initio method to calculate the Raman spectra. The characteristic vibrational wavenumber of the Al-F symmetric stretching vibrational modes of the [AlF_4_]^−^_n_ clusters were then estimated accordingly.

**Figure 10:**
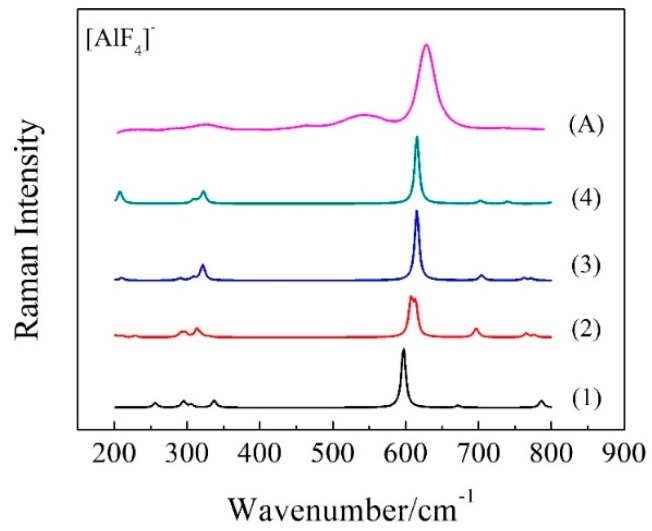
Calculated Raman spectra of various model clusters (shown in Figure 9), corrected for frequency shift with a factor of 0.90, and experimental spectrum of molten KAlF_4_ (Spectrum A).

**Figure 11:**
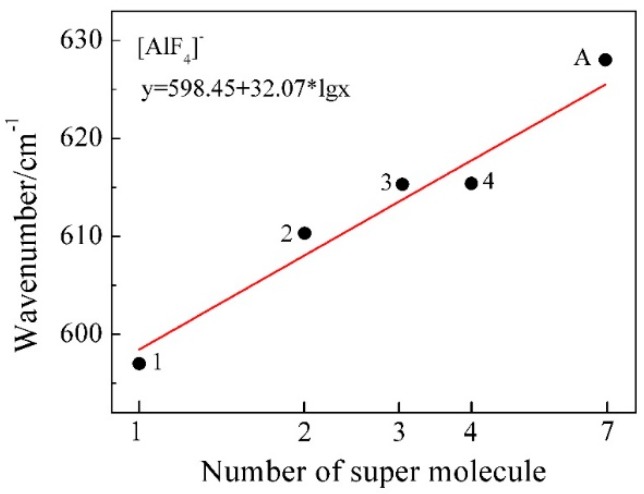
Calculated characteristic Raman wavenumber of Al-F symmetric stretching vibrational modes as a function of log n (the number of molecules in [AlF_4_]^−^_n_ cluster). The reference point A in the figure represents the experimental characteristic wavenumber of molten KAlF_4_.

The restricted Hartree-Fock calculation method using a 6-31G (d) [31,32] basis set and was applied for geometry optimization and simulation of Raman vibrational modes of possible model clusters in the melt. When the calculated Raman spectra are compared with the experimental results, corrections are often needed for the intensity and frequency due to the effect of experimental temperature and the laser wavelength [33]. The wavenumbers of the calculated Raman spectra in Figure 10 have been corrected using a scaling factor of 0.90 [34] to better represent the experimental Raman spectrum. The characteristic wavenumber of Al-F symmetric stretching vibrational modes was further estimated from Figure 10 and compared with the experimental and reported values in Table 4. The local structure environment of a crystal is believed to have brought an impact on the structure in of the melt when it is melted. Therefore it was assumed that there are six [AlF_4_]^−^ tetrahedra in the neighbor of an isolated [AlF_4_]^−^ tetrahedron in the melt according to the crystal KAlF_4_ structure in KAlF_4_. In Figure 11, the characteristic wavenumber of the symmetric stretching vibrational modes of a particular model cluster was plotted in a log scale as a liner function of the number of molecules in the [AlF_4_]^−^_n_ cluster. As the number of molecules in the clusters increased, the characteristic wavenumber of the Al-F symmetric stretching vibrational mode of [AlF_4_]^−^_n_ appeared to increase. The symmetric stretching vibrational wavenumber of the model cluster with seven molecules of [AlF_4_]^−^ was therefore estimated to be 625.6 cm^−1^, which was in good agreement with the values observed in the experiment and reported in the literature [15,35,36,37].

As shown in Figure 7, the Raman band of 628 cm^−1^, which was attributed to the symmetric stretching vibrations of Al^IV^-F_nb_ of the [AlF_4_]^−^ tetrahedron, appeared at 863 K. Simultaneously, the intensity of the Raman band of 628 cm^−1^ increased while the intensity of the Raman band of 536 cm^−1^ decreased. The six-coordinated Al atom began to turn into four-coordinated. At 893 K, KAlF_4_ had been completely melted, revealing the symmetric stretching vibrations of Al^IV^-F_nb_ of the [AlF_4_]^−^ tetrahedron and little symmetric stretching vibrations of Al^VI^-F_nb_ of the [AlF_6_]^3−^ octahedron were in the melt.

## 4. Conclusions

The structure evolution of KAlF_4_ was investigated by in situ high temperature X-ray diffraction and Raman spectroscopy. The major vibrational modes of temperature dependent Raman spectra of KAlF_4_ were assigned based on the calculated spectra by both density functional theory and quantum chemistry ab initio methods.

Below the melting point, solid KAlF_4_ was found to present in two different polymorph phases at room and high temperature. Both polymorph KAlF_4_ phases are characterized by a layered structure consisting of K^+^ and [AlF_6_]^3−^ octahedra, where each [AlF_6_]^3−^ octahedron equivalently shares four corners with other four [AlF_6_]^3−^ octahedra along the layer. While the room temperature phase is in the tetragonal cell (space group P4/mbm), the high temperature phase undergoes a monoclinic distortion (space group P2_1_/m) together with an ordering of the K atoms.

The layer structure decomposed with increasing temperature and finally turned to [AlF_4_]^−^ tetrahedra when the temperature exceeds the melting point. It was hence indicated that in molten KAlF_4_, there is a principal amount of [AlF_4_]^−^ and a small amount of [AlF_6_]^3−^. This was further confirmed by quantum chemistry ab initio simulations.

## Figures and Tables

**Table 1:** The assignment of major room temperature vibration modes of crystalline KAlF_4_.

Wavenumber		Vibrational Modes	Type of Vibration
υ_exp_ (cm^−1^)	υ_cal_ (cm^−1^)		
228	227.6	*E_g_*	γ (Al^VI^-F_nb_) ^1^
547	547.4	*A*_1*g*_	υ_s_ (Al^VI^-F_nb_) ^1^

^1^ γ and υ represent the shearing and stretching vibrations, respectively. Subscript s represents the symmetric vibration. Al^VI^ denotes a six-coordinated Al atom.

**Table 2:** The assignment of major vibration modes of high temperature phase of KAlF_4_.

Wavenumber		Vibrational Modes	Type of Vibration
υ_exp_ (cm^−1^)	υ_cal_ (cm^−1^)		
229	193.9	*B_g_*	γ (Al^VI^-F_nb_) ^1^
319	335.9	*A_g_*	γ (Al^VI^-F_nb_) ^1^
481	474.5	*A_g_*	γ (Al^VI^-F_nb_) ^1^
539	539.5	*A_g_*	υ_s_ (Al^VI^-F_nb_) ^1^

^1^ γ and υ represent the shearing and stretching vibrations, respectively. Subscript s represents the symmetric vibration. Al^VI^ denotes a six-coordinated Al atom.

**Table 3:** Structure parameters of room and high temperature phases of KAlF_4_.

Parameter		Compound	
		Room Temperature Phase	High Temperature Phase
Crystal System		tetragonal	monoclinic
Space Group		P4/mbm	P2_1_/m
Cell Length/Å	a	5.122	6.542
	b	5.122	7.195
	c	6.288	7.177
Cell Angle/°	α	90.000	90.000
	β	90.000	108.478
	γ	90.000	90.000
Bond Length/Å	Al^VI^-F_b_	1.733	1.829, 1.800
	Al^VI^-F_nb_	1.674	1.769, 1.768
Bond Angle/°	∠F_b_-Al^VI^-F_b_	90.000	89.307, 90.693, 90.307, 89.693
	∠F_b_-Al^VI^-F_nb_	90.000	90.958, 91.871, 88.129, 89.042, 91.204, 92.520, 87.480, 88.796
	∠Al^VI^-F_b_-Al^VI^	167.476	170.788(2), 159.189, 158.912

**Table 4:** Calculated, experimental and reported Raman wavenumber of [AlF_4_]^−^ clusters.

Clusters	Wavenumber (cm^−1^)								
	Number of Molecules						Exp.	Ref. 10 (Cal.)	Ref. 35 (Exp.)
	1	2	3	4	…	n = 7			
[AlF_4_]^−^_n_	597.0	610.3	615.3	615.4	…	625.6	628	631	622

## Supplementary Materials

The following are available online at http://www.mdpi.com/1996-1944/11/10/1846/s1, Table S1: The assignment of all calculated room temperature Raman vibration modes of crystalline KAlF_4_, Table S2: The assignment of all calculated Raman vibration modes of high temperature phase of KAlF_4_.
