# Supplementary material for: Temperature Dependent Micro-Structure of KAlF4 from Solid to Molten States
Source: Materials (Basel). 2018 Sep 27;11(10):1846. doi: 10.3390/ma11101846 (PMC6213106; doi:10.3390/ma11101846)
Supplement: Supplementary file 1 [file materials-11-01846-s001.pdf]

Table S1. The assignment of all calculated room temperature Raman vibration modes of crystalline $KAlF_4$.

Wavenumber (cm^{-1})	Vibrational Modes	Type of Vibration	Raman Intensity Active
90.1	A_{1g}	δ (Al^{VI}-F-Al^{VI}) [1]	0.20
113.6 [2]	E_g	γ (Al^{VI}-F_{nb}) [1]	0.00036
227.6 [2, 3]	E_g	γ (Al^{VI}-F_{nb}) [1]	0.38
280.2	B_{2g}	$υ_s$ (Al^{VI}-F-Al^{VI}) [1]	0.0063
408.3	B_{1g}	δ (Al^{VI}-F-Al^{VI}) [1]	0.00002
458.4 [2]	E_g	δ (Al^{VI}-F-Al^{VI}) [1]	0.0060
547.4 [3]	A_{1g}	$υ_s$ (Al^{VI}-F_{nb}) [1]	5.52

[1] γ, δ and υ represent the shearing, bending and stretching vibrations, respectively. Subscript s represents the symmetric vibration. Al^{VI} denotes a six-coordinated Al atom.
[2] There are two modes of degeneracy.
[3] The one is as a major vibration mode shown in the article.

Table S2. The assignment of all calculated Raman vibration modes of high temperature phase of $KAlF_4$.

Wavenumber (cm^{-1})	Vibrational Modes	Type of Vibration	Raman Intensity Active
-349.3	B_g	—	0
124.0	A_g	Cation vibration	0.0097
128.4	A_g	Cation vibration	0.31
153.1	A_g	Cation vibration	0.055
160.6	B_g	Cation vibration	0.00023
193.9 [2]	B_g	γ (Al^{VI}-F_{nb}) [1]	0.64
206.2	A_g	δ (Al^{VI}-F-Al^{VI}) [1]	0.0070
209.4	B_g	γ (Al^{VI}-F_{nb}) [1]	0.0090
224.8	A_g	γ (Al^{VI}-F_{nb}) [1]	0.018
225.1	B_g	γ (Al^{VI}-F_{nb}) [1]	0.074
252.3	A_g	δ (Al^{VI}-F-Al^{VI}) [1]	0.0075
282.8	B_g	$υ_s$ (Al^{VI}-F-Al^{VI}) [1]	0.00029
285.1	A_g	δ (Al^{VI}-F-Al^{VI}) [1]	0.010
291.8	B_g	δ (Al^{VI}-F-Al^{VI}) [1]	0.0020
293.1	A_g	δ (Al^{VI}-F-Al^{VI}) [1]	0.0010
298.7	A_g	δ (Al^{VI}-F-Al^{VI}) [1]	0.00093
331.6	B_g	γ (Al^{VI}-F_{nb}) [1]	0.20
335.9 [2]	A_g	γ (Al^{VI}-F_{nb}) [1]	0.62
375.4	B_g	$υ_s$ (Al^{VI}-F-Al^{VI}) [1]	0.00054
432.2	A_g	$υ_s$ (Al^{VI}-F-Al^{VI}) [1]	0.00011
433.8	B_g	$υ_s$ (Al^{VI}-F-Al^{VI}) [1]	0.00062
453.1	A_g	δ (Al^{VI}-F-Al^{VI}) [1]	0.010
471.4	B_g	δ (Al^{VI}-F-Al^{VI}) [1]<br>γ (Al^{VI}-F_{nb}) [1]	0.00047
474.5 [2]	A_g	γ (Al^{VI}-F_{nb}) [1]	0.44
477.0	A_g	δ (Al^{VI}-F-Al^{VI}) [1]	0.011

478.8	A_g	γ (Al^{VI}-F_{nb}) [1]<br>δ (Al^{VI}-F-Al^{VI}) [1]<br>γ (Al^{VI}-F_{nb}) [1]	0.19
539.5 [2]	A_g	υ_s (Al^{VI}-F_{nb}) [1]	11.59
541.6	B_g	υ_s (Al^{VI}-F_{nb}) [1]	0.0082
547.0	A_g	υ_s (Al^{VI}-F_{nb}) [1]	0.0055
553.1	B_g	υ_s (Al^{VI}-F_{nb}) [1]	0.0018

[1] γ, δ and υ represent the shearing, bending and stretching vibrations, respectively. Subscript s represents the symmetric vibration. Al^{VI} denotes a six-coordinated Al atom.

[2] The one is as a major vibration mode shown in the article.
